# A real-world analysis of hybrid CDA and ACDF compared to multilevel ACDF

**DOI:** 10.1186/s12891-023-06284-2

**Published:** 2023-03-14

**Authors:** Kee D Kim, Domagoj Coric, Armen Khachatryan, Brenna L Brady, Timothy Lillehaugen, Mike McCormack, William B Dolman, Richard Ditto

**Affiliations:** 1 UC Davis Health, 4860 Y Street Suite, Sacramento, CA 3740, 95817 USA; 2 Atrium Musculoskeletal Institute, Spine Division, Carolina Neurosurgery and Spine Associates, 225 Baldwin Avenue Charlotte, Charlotte, NC 28204 USA; 3 Orthopedic Spine Surgery, The Disc Replacement Center, 3584 West 9000 South #209, Salt Lake City, UT 84088 USA; 4 Merative, 75 Binney St., Cambridge, MA 02142 USA; 5 Zimmer Biomet, 1800 W Center Street, Warsaw, IN 46580 USA; 6 ZimVie Spine, 10225 Westmoor Drive, Westminster, CO 80021 USA

**Keywords:** Cervical spondylosis, Cervical arthroplasty, Anterior cervical fusion, Adjacent segment disease, Hybrid surgery, Intervertebral disc surgery, Spinal cord diseases

## Abstract

**Background:**

Multilevel anterior cervical discectomy and fusion (mACDF) is the gold standard for multilevel spinal disease; although safe and effective, mACDF can limit regular spinal motion and contribute to adjacent segment disease (ASD). Hybrid surgery, composed of ACDF and cervical disc arthroplasty, has the potential to reduce ASD by retaining spinal mobility. This study examined the safety of hybrid surgery by utilizing administrative claims data to compare real-world rates of subsequent surgery and post-procedural hospitalization within populations of patients undergoing hybrid surgery versus mACDF for multilevel spinal disease.

**Methods:**

This observational, retrospective analysis used the MarketScan Commercial and Medicare Database from July 2013 through June 2020. Propensity score matched cohorts of patients who received hybrid surgery or mACDF were established based on the presence of spinal surgery procedure codes in the claims data and followed over a variable post-period. Rates of subsequent surgery and post-procedural hospitalization (30- and 90-day) were compared between hybrid surgery and mACDF cohorts.

**Results:**

A total of 430 hybrid surgery patients and 2,136 mACDF patients qualified for the study; average follow-up was approximately 2 years. Similar rates of subsequent surgery (Hybrid: 1.9 surgeries/100 patient-years; mACDF: 1.8 surgeries/100 patient-years) were observed for the two cohorts. Hospitalization rates were also similar across cohorts at 30 days post-procedure (Hybrid: 0.67% hospitalized/patient-year; mACDF: 0.87% hospitalized/patient-year). At 90 days post-procedure, hybrid surgery patients had slightly lower rates of hospitalization compared to mACDF patients (0.23% versus 0.42% hospitalized/patient-year; p < 0.05).

**Conclusions:**

Findings of this real-world, retrospective cohort study confirm prior reports indicating that hybrid surgery is a safe and effective intervention for multilevel spinal disease which demonstrates non-inferiority in relation to the current gold standard mACDF. The use of administrative claims data in this analysis provides a unique perspective allowing the inclusion of a larger, more generalizable population has historically been reported on in small cohort studies.

## Introduction

Anterior cervical discectomy and fusion (ACDF) has been the gold standard for treating spondylosis and degenerative disc disease [1]. The procedure, which involves decompression and fusion, is associated with very positive clinical outcomes [2]. Although effective, ACDF results in decreased overall range of motion due to loss of segmental motion at the site of the fusion, which can place increased stress on adjacent vertebrae and alter mobility. Additional stress at the adjacent levels from ACDF has been postulated to promote adjacent segment disease (ASD) that is characterized by symptomatic degeneration of nearby vertebral discs. Treatment of ASD often requires additional fusion of adjacent vertebrae. Estimates indicate a 10-year post-fusion prevalence of symptomatic ASD of up to 26% following ACDF with more than two-thirds of those patients requiring additional operative procedures [1–3]. In contrast to ACDF, cervical disc arthroplasty (CDA) helps to preserve range of motion by using artificial disc implants to replace the damaged disc. Although ACDF is safe, effective, and remains the current standard, studies have reported a 2- to 4-fold reduction in adjacent segment pathology with motion preserving CDA procedures, providing a potential benefit of this newer technology [1, 4−5].

Preservation of spinal motion is especially critical in multilevel procedures. The presence of multilevel ACDF (mACDF) with a longer lever arm further restricts regular spinal motion compared to a single level fusion and thus has a greater potential to alter mobility. As a result, multilevel fusions place even greater stress on adjacent segments compared to single level procedures increasing the potential for ASD. To combat loss of motion and to reduce the risk of ASD in multilevel procedures, hybrid surgery (CDA with ACDF) has been proposed [2]. Although hybrid surgery has become more popular, there remains limited evidence regarding its efficacy compared to ACDF [1, 6]. To date, biomechanical studies have reported hybrid surgery is associated with improved range of motion compared to mACDF [7–9]. Hybrid surgery has also been found to be safe in the select cases in which it has been used [2].

However, data regarding comparative patient outcomes with hybrid surgery versus mACDF remain scarce, and there is limited information on real-world outcomes. To address this data gap, this retrospective analysis used administrative claims data to examine and compare rates of subsequent surgery and hospitalization within matched groups of patients receiving mACDF or hybrid surgery for multilevel spinal disease. Although clinical data are limited in administrative claims databases, the large population in conjunction with the availability of comprehensive records of patient’s full patient healthcare experience, including both encounters and procedures, allows for a unique, real-world assessment of spinal surgery outcomes in a more generalizable population than may be present in other clinical or observational analyses conducted to date.

## Methods

### Study Design and Data source

This study utilized the MarketScan Commercial and Medicare Supplemental Databases, composed of de-identified patient-level administrative claims data, from July 1, 2013 through June 30, 2020. The Commercial and Medicare Supplemental databases contain all healthcare claims (inpatient, outpatient, and outpatient pharmacy) for patients covered by commercial or Medicare Supplemental insurance plans offered through a former or current employer. Both supplemental insurance and Medicare paid portions of claims are represented for patients with Medicare Supplemental insurance. All study data were obtained using International Classification of Diseases, 9th and 10th Revision, Clinical Modification (ICD-9-CM and ICD-10-CM) codes, Current Procedural Terminology 4th edition codes, Healthcare Common Procedure Coding System codes, and National Drug Codes.

### Patient selection and cohort assignment

The sample was composed of adults with evidence of mACDF (≥ 2 ACDF procedures) or hybrid surgery (≥ 1 ACDF and ≥ 1 CDA procedure). The first qualifying spinal surgery procedure (ACDF or hybrid) served as the study index date. Patients were required to have ≥ 6-months of eligibility prior to index; post-index follow-up was variable in length and ending at the end of continuous eligibility or the end of study data, whichever occurred first. Imposition of continuous eligibility over patient follow-up ensured that all healthcare encounters (e.g., hospitalizations, spinal procedures) were captured. Spinal surgeries could have occurred on the same day (primary multilevel surgery) or via procedures on independent days (secondary multilevel surgery). In the case of secondary hybrid surgeries, the ACDF procedure had to have occurred first to evaluate the potential benefit of secondary hybrid fusion versus mACDF after primary fusion.

To define the total number of affected vertebrae, unique procedures occurring during the post-index period were defined. The first instance of each CPT code (ACDF: 22551, 22552; CDA: 22856, 22858) that was present on one day was counted as one procedure (i.e., one level surgery). Multiple procedures on the same day (i.e., multilevel primary surgery) were identified as two unique codes on the same day (e.g., 22551 and 22552) or the presence of the same code on multiple claims occurring on the same day, provided that the code appeared at least once without any modifiers (first procedure) and at least once with a ‘59’ modifier indicating an additional procedure. The surgical index date was the date of the first procedure that qualified the patient as having multilevel surgery. For patients with primary surgery, this date was the same as the index date; for patients with secondary surgery the surgical index date occurred after the study index date.

Patients eligible for the hybrid surgery or mACDF cohorts were classified based on their qualifying procedures:

- 2-level Primary Surgery Cohort – patient had two qualifying surgeries occurring on the same date.
- 3+-level Primary Surgery Cohort – patient had three or more qualifying surgeries occurring on the same date.
- Secondary Surgery Cohort – patient had at least two qualifying surgeries on different days. By definition, patients had a single surgery on the first day; they were allowed to have more than one qualifying surgery on the second day. Due to sample size, this cohort was not split into 2-level and 3+-level groups.

Propensity score matching was used to ensure comparability of baseline demographics and clinical characteristics between mACDF and hybrid surgery cohorts. Patients in the hybrid surgery cohort were matched to patients in the mACDF cohort at up to a 1:5 ratio. Stratified matching based on sub-cohorts was used and propensity score covariates included age, sex, duration of follow-up, mean Charlson Comorbidity Index, index year, region of residence, and plan type (2-level primary sub-cohort match only).

### Study period, outcomes, and analysis

Patients were followed over the 6-months prior to study index (pre-period) through the end of continuous eligibility or the end of study data (variable length post-period). The post-period was further divided into procedural episodes to facilitate an episode level analysis of subsequent surgeries and post-procedural hospitalizations. Procedural episodes initiated at the surgical index date and continued until evidence of another surgery that added at least one surgical level (e.g., patient moved from two affected intervertebral spaces to three) or the end of follow-up. The addition of a surgical level was defined as the presence of a surgical day where the patient evidenced a greater number of claims for spinal procedures (based on the above counting logic) than surgical revision codes (CPT: 22830, 22849, 22855, 22861, 22864, 0095T, 0098T). Days that met this criterion were considered to increase the number of surgical levels regardless of whether a revision surgery code was present; all other combinations were considered revision surgeries that did not increase the total number of surgical levels and thus did not end the procedural episode. This approach allowed for revision surgeries that did not increase the number of surgical levels to occur during procedural episodes.

Demographics were assessed on index, while baseline clinical characteristics were examined over the pre-period. The primary study outcomes, the rate of subsequent surgery and all-cause or spinal surgery-related post-procedure hospitalizations at 30- and 90-days, were assessed over the first two procedural episodes in the post-period; due to low numbers of patients with > 2 episodes the episode analysis was not extended beyond the first two procedural episodes. To adjust outcomes for patient observation time, primary outcomes were calculated over the defined period (e.g., 30 days, 90 days, procedural episode) or until patient censoring, defined as the first of: the end of the defined period, the end of study data, or end of continuous eligibility. All analyses were conducted using WPS version 4.1 (World Programming, United Kingdom).

Categorical variables were presented as the count and percentage; continuous variables were summarized by providing the mean, standard deviation, median, and range. Rates of subsequent surgery were reported as incidence rates (events/patient-years), while post procedural hospitalizations were reported as the percent of patients hospitalized per patient observation year, to account for variable follow-up. Differences between overall hybrid surgery and mACDF cohorts were examined along with differences between the three sub-cohorts within each. Chi-square tests were used to evaluate the statistical significance of differences for categorical variables; t-tests and ANOVA were used for continuous variables. A critical value of 0.05 was specified *a priori* as the threshold for statistical significance.

## Results

### Study sample

After propensity score matching there were 430 hybrid surgery patients and 2,136 mACDF patients included in the analysis. The majority of the sample (61%) had 2-level primary surgery as the qualifying surgery, followed by 30% with 3+-level primary surgery, and 9% with secondary surgery (Table 1). Patients were middle-aged with a mean age of 49 ± 9 years for both cohorts; sex distribution was roughly equal (Table 2). Average post-index follow-up was 687 ± 519 days for the hybrid surgery cohort (range 2 to 2,287 days) and 718 ± 564 days for the mACDF cohort (range 1 to 2,368 days). Minor imbalances in urbanicity (Hybrid: 91% vs. mACDF: 83%) and presence of other cervical disc disorders in the baseline period (Hybrid: 61.2% vs. mACDF: 47.1%) persisted in the matched cohorts (Table 2).

**Table 1:** Sample Attrition

Inclusion/Exclusion Criteria	Hybrid Surgery		mACDF	
	N	%	N	%
Evidence of hybrid surgery or multilevel ACDF between January 1, 2014 and June 30, 2020 *(first surgical procedure serves as the index date)*	558	100%	61,625	100%
AND Age 18 or older on index	558	100%	61,587	99.90%
AND Continuous enrollment for ³6 months prior to index	444	79.60%	46,775	75.90%
AND No evidence of ACDF or CDA procedures prior to index	441	79.00%	46,464	75.40%
AND No evidence of other related spinal surgeries in the pre-period (Total Pre-Match Sample)	430	77.10%	45,818	74.30%
Pre-match Sub-cohorts				
2-level primary	262	60.90%	43,448	94.80%
3+-level primary	131	30.50%	1540	3.40%
Secondary	37	8.60%	830	1.80%
Post-match Samples				
Total Cohort	430	100%	2,136	100%
2-level primary	262	60.90%	1,310	61.30%
3+-level primary	131	30.50%	641	30.00%
Secondary	37	8.60%	185	8.70%

**Table 2:** Characteristics of hybrid surgery and multilevel ACDF cohorts

	Hybrid Surgery ALL Patients		mACDF ALL Patients		*Standardized Difference* ^*2*^
	N=	430	N=	2,136	
	N/Mean	%/SD	N/Mean	%/SD	
**Age (Mean, SD)**	48.8	9.2	49.2	9.2	4.0
Median	49.0		49.0		
Range	24	80	18	92	
**Sex (N, %)**					
Male	211	49.1%	1,047	49.0%	0.1
Female	219	50.9%	1,089	51.0%	0.1
**Geographic region (N, %)**					
Northeast	71	16.5%	338	15.8%	1.9
North Central	81	18.8%	396	18.5%	0.8
South	197	45.8%	993	46.5%	1.4
West	76	17.7%	393	18.4%	1.9
Unknown	5	1.2%	16	0.7%	4.3
**Population Density (N, %)**					
Urban	392	91.2%	1,773	83.0%	24.5
Rural	33	7.7%	348	16.3%	26.8
Unknown	5	1.2%	15	0.7%	4.8
**Insurance plan type** ^**1**^ **(N, %)**					
Comprehensive/indemnity	13	3.0%	67	3.1%	0.7
EPO/PPO	262	60.9%	1,258	58.9%	4.2
POS/POS with capitation	38	8.8%	202	9.5%	2.1
HMO	43	10.0%	200	9.4%	2.2
CDHP/HDHP	68	15.8%	368	17.2%	3.8
Other/Unknown	6	1.4%	41	1.9%	4.1
**Index year (N, %)**					
2014	66	15.3%	312	14.6%	2.1
2015	84	19.5%	446	20.9%	3.4
2016	60	14.0%	308	14.4%	1.3
2017	87	20.2%	462	21.6%	3.4
2018	73	17.0%	345	16.2%	2.2
2019	59	13.7%	254	11.9%	5.5
2020	1	0.2%	9	0.4%	3.3
**Charlson Comorbidity Index (CCI) (Mean, SD)**	0.5	0.9	0.4	0.9	1.7
Range	0	8	0	8	
**Spinal Disease Conditions (N, %)**					
Cervical spondylosis	229	53.3%	1,211	56.7%	6.9
Degenerative disc disease	182	42.3%	866	40.5%	3.6
Other cervical disc disorders	263	61.2%	1,006	47.1%	28.5
**Other Comorbid Conditions (N, %)**					
Osteoporosis	4	0.9%	23	1.1%	1.5
Radiculopathy or myelopathy	263	61.2%	1,261	59.0%	4.3
Pain conditions	337	78.4%	1,645	77.0%	3.3
* Neck*	307	71.4%	1,492	69.9%	3.4
* Shoulder*	98	22.8%	518	24.3%	3.4
* Arm*	64	14.9%	313	14.7%	0.6
**Duration of Follow-up (Mean, SD)**	687.3	519.0	718.4	563.7	5.7
Range	2	2287	1	2368	

^1^ EPO: Exclusive provider organization; PPO: Preferred provider organization; HMO: Health maintenance organization; POS: Point of service; CDHP: Consumer-driven health plan; HDHP: High deductible health plan

^2^ Standardized difference is the measure of effect size used to assess balance between cohorts during propensity score matching; values > 10 indicate imbalance for the specific covariate of interest

mACDF: multilevel anterior discectomy and fusion

### Subsequent surgery

The 430 hybrid surgery patients contributed 436 procedural episodes to the subsequent surgery analysis, while the 2,136 mACDF patients contributed 2,180 episodes. Rates of subsequent surgery accounting for follow-up time were comparable across hybrid surgery and mACDF cohorts (Table 3). The hybrid surgery cohort had a rate of 1.9 surgeries/100 patient-years compared to a rate of 1.8 surgeries/100 patient-years for the mACDF cohort. The 2-level primary cohorts tended to have the lowest rates of subsequent surgery at 1.2 and 1.7 surgeries/100 patient-years in the hybrid surgery and mACDF cohorts, respectively. Rates of subsequent surgery increased slightly in the 3+-level primary cohorts with the hybrid surgery and mACDF patients evidencing subsequent surgery rates at 2.2 and 1.8 surgeries/100 patient-years, respectively. The secondary surgery sub-cohorts had the highest rates of subsequent surgery with 5.5 surgeries/100 patient-years in the hybrid surgery cohort and 1.9 surgeries/100 patient-years in the mACDF cohort.

**Table 3:** Subsequent surgery and hospitalization in hybrid CDA + ACDF vs. multi-level ACDF.

	Full Sample					Primary 2-Level Surgery					Primary 3+-Level Surgery					Secondary Surgery				
	Hybrid Surgery ALL Episodes		mACDF ALL Episodes		p^1^	Hybrid Surgery ALL Episodes		mACDF ALL Episodes		p^1^	Hybrid Surgery ALL Episodes		mACDF ALL Episodes		p^1^	Hybrid Surgery ALL Episodes		mACDF ALL Episodes		p^1^
	N=	436	N=	2,180		N=	264	N=	1,337		N=	132	N=	654		N=	40	N=	189	
**Subsequent Surgery**																				
**Duration of Follow-up (Mean, SD)**	673.8	514.5	704.1	559.0		694.6	523.0	726.6	570.5		636.7	495.2	660.1	536.1		658.6	526.2	696.9	559.0	
**Procedure Episodes**																				
Episodes with evidence of a subsequent surgery (N, %)	9	2.1%	46	2.1%		3	1.1%	29	2.2%		3	2.3%	13	2.0%		3	7.5%	4	2.1%	
Rate of subsequent surgery (per 100 patient years)	1.9	-	1.8	-		1.2	-	1.7	-		2.2	-	1.8	-		5.5	-	1.9	-	
**30-Days Hospitalization**																				
**All-cause Hospitalization**																				
Episodes with a patient admission (N, %)	20	4.6%	99	4.5%		8	3.0%	64	4.8%		7	5.3%	30	4.6%		5	12.5%	5	2.6%	< 0.01
Days to hospitalization (Mean, SD)	7.0	6.5	8.9	8.1		3.5	2.7	8.8	8.3		7.0	7.5	8.7	7.6		12.6	6.2	11.0	10.1	
Median	5.5	-	7.0	-		3.0	-	6.0	-		4.0	-	7.0	-		13.0	-	7.0	-	
Range	1	22	1	30		1	6	1	30		1	21	1	28		6	22	2	25	
% hospitalized (per 1 patient year)	0.67	-	0.87	-		0.43	-	0.90	-		0.87	-	0.98	-		1.64	-	0.33	-	< 0.05
**Spinal-Surgery Specific** **Hospitalization** ^2^																				
Episodes with a patient admission (N, %)	16	3.7%	76	3.5%		8	3.0%	48	3.6%		5	3.8%	24	3.7%		3	7.5%	4	2.1%	
Days to hospitalization (Mean, SD)	7.6	6.7	8.3	7.7		4.8	4.6	8.0	7.8		9.4	7.8	8.2	7.3		12.0	8.7	13.0	10.4	
Median	6.0	-	6.0	-		3.5	-	6.0	-		9.0	-	6.5	-		8.0	-	12.5	-	
Range	1	22	1	30		1	14	1	30		1	21	1	28		6	22	2	25	
% hospitalized (per 1 patient year)	0.46	-	0.61	-		0.38	-	0.56	-		0.48	-	0.82	-		0.96	-	0.26	-	
**90-Days Hospitalization**																				
**All-cause Hospitalization**																				
Episodes with a patient admission (N, %)	20	4.6%	140	6.4%		8	3.0%	87	6.5%	< 0.05	7	5.3%	45	6.9%		5	12.5%	8	4.2%	< 0.05
Days to hospitalization (Mean, SD)	7.0	6.5	24.5	27.2	< 0.01	3.5	2.7	23.5	27.1	< 0.05	7.0	7.5	26.2	28.0		12.6	6.2	26.4	26.3	
Median	5.5	-	11.0	-		3.0	-	9.0	-		4.0	-	11.0	-		13.0	-	21.5	-	
Range	1	22	1	88		1	6	1	88		1	21	1	88		6	22	2	80	
% hospitalized (per 1 patient year)	0.23	-	0.42	-	< 0.05	0.14	-	0.42	-	< 0.05	0.33	-	0.48	-		0.57	-	0.18	-	< 0.05
**Spinal-Surgery Specific** **Hospitalization** ^2^																				
Episodes with a patient admission (N, %)	16	3.7%	106	4.9%		8	3.0%	65	4.9%		5	3.8%	35	5.4%		3	7.5%	6	3.2%	
Days to hospitalization (Mean, SD)	7.6	6.5	22.9	27.2	< 0.05	4.8	2.7	20.8	27.1		9.4	7.5	25.6	28.0		12.0	6.2	29.5	26.3	
Median	6.0	-	9.0	-		3.5	-	9.0	-		9.0	-	9.0	-		8.0	-	21.5	-	
Range	1	22	1	89		1	14	1	88		1	21	1	89		6	22	2	80	
% hospitalized (per 1 patient year)	0.15	-	0.21	-		0.13	-	0.19	-		0.16	-	0.28	-		0.33	-	0.09	-	

mACDF: multilevel anterior cervical discectomy and fusion; SD: standard deviation

^1^ The lack of a p-value figure indicates comparison was not significant

^2^ The presence of spinal surgery- or spinal disease-related procedure or diagnosis codes on inpatient claims will be used to define spinal surgery-specific readmissions

### Hospitalizations

In the first 30 days following the first or second procedural episode there were 20 (4.6%) and 99 (4.5%) episodes with ≥1 all-cause hospitalization in the hybrid surgery cohort and mACDF cohorts respectively. A majority (77.3%) of these hospitalizations were spinal surgery-related, with 16 (3.7%) episodes in the hybrid surgery cohort and 76 (3.5%) episodes in the mACDF cohort. Overall, the average time to all-cause hospitalization (7.0 vs. 8.9 days) and rate of all-cause hospitalization (0.67% vs. 0.87% hospitalized/patient-year) were similar between the hybrid surgery and mACDF cohorts respectively. Among the sub-cohorts the only difference between the hybrid surgery and mACDF patients was observed between the secondary surgery groups, with the secondary hybrid surgery sub-cohort evidencing a higher frequency of episodes with an all-cause hospitalization (12.5% vs. 2.6%; p < 0.01) and a higher rate of 30-day all-cause hospitalization (1.64% vs. 0.33% hospitalized/patient-year; p < 0.05) compared to the secondary mACDF sub-cohort (Table 3).

At 90 days post-procedure there were 20 (4.6%) episodes with ≥1 hospitalization in the hybrid surgery cohort, indicating no additional episodes with a hospitalization past 30 days post-procedure. There were 140 (6.4%) episodes with a hospitalization in the 90 days following the surgical procedure in the mACDF cohort. Again, the majority of hospitalizations at 90 days were classified as spinal surgery related (Table 3). The hybrid surgery cohort had a significantly shorter time to hospitalization at 90 days both for all-cause (7.0 vs. 24.5 days; p < 0.01) and spinal surgery-related hospitalizations (7.6 vs. 22.9 days; p < 0.05) compared to the mACDF cohort. Consistent with the lack of new episodes with a hospitalization post-30 days, the rate of all-cause hospitalization at 90 days was lower for the hybrid surgery cohort compared to the mACDF cohort (0.23% vs. 0.42% hospitalized/patient-year; p < 0.05). Within the sub-cohorts, the frequency of episodes with a 90-day all-cause hospitalization (3.0% vs. 6.5%; p < 0.05) and the rate of all-cause 90-day hospitalizations (0.14% vs. 0.42% hospitalized/patient-year; p < 0.05) were significantly lower in the 2-level primary hybrid surgery cohort compared to the 2-level primary mACDF cohort. Conversely, the frequency of episodes with a 90-day all-cause hospitalization (12.5% vs. 4.2%; p < 0.05) and rate of 90-day all-cause hospitalizations (0.57% vs. 0.18% hospitalized/patient-year; p < 0.05) were higher in the secondary hybrid surgery cohort compared to the secondary mACDF cohort.

## Discussion

The goal of hybrid surgery is to provide the most suitable treatment for each cervical disc, making the procedure appropriate for select patients with different types of disease and different degrees of degeneration at adjacent levels. This retrospective analysis of administrative claims-based data utilized the MarketScan Commercial and Medicare Supplemental Databases to investigate rates of subsequent surgery and hospitalization between patients undergoing hybrid surgery or mACDF to treat multilevel cervical degenerative disc disease. This study is one of the largest known analyses of real-world outcomes in patients receiving hybrid surgery to date.

Overall, our results demonstrate non-inferiority of hybrid surgery compared to the current standard of care as assessed via claims-based rates of subsequent surgery and post-procedural all-cause and spinal surgery related hospitalization. Within our study sample, we found no evidence of increased rates of subsequent surgery following hybrid procedures when compared to mACDF, suggesting no notable risk of CDA failure in these multilevel constructs. Our use of real-world administrative claims data is unique and adds to the current literature by providing outcomes within a larger, more nationally representative population of patients than may have been able to be included in smaller clinical or observational studies. These results thus help to extend prior, more clinically based findings to a broader, more generalizable patient sample that may be more reflective of patients receiving multi-level spinal surgery in the real-world.

Previous clinical studies found hybrid surgery to be associated with greater postoperative C2-C7 range of motion (ROM), reduced ROM at the adjacent levels, reduced need for subsequent surgery, and faster return to work than mACDF [1]. Although our results do not directly address these benefits of hybrid surgery, our finding of non-inferiority in regard to subsequent surgery and post-procedural hospitalization is not out of line with these findings. In theory, when fewer segments are fused, as is done in hybrid surgery versus mACDF, there is less compensatory activity and subsequent degeneration in adjacent segments. Fusion of multiple segments in mACDF can result in more pseudoarthrosis, lower fusion rates, more graft subsidence, and increased risk of subsequent surgery compared to single level fusions. Towards this, Swank et al. reported that the likelihood of pseudoarthrosis increased from 10% in 1-level surgery to 44% and 45% in 2-level and 3-level surgeries respectively [10]. Brodke and Zdeblick reported a fusion rate in 1-level ACDF as high as 97%, whereas the fusion rate in 3-level ACDF decreased to 83%, while Zigler et al. reported fusion rates at 2 years were 89.3% in 1-level ACDF vs. 79.8% in 2-level ACDF [11–13]. Similarly, rates of subsequent surgery at the index fusion level were reported to be approximately 1.8-fold higher at two years post-procedure, and 1.5-fold higher at 5 years post procedure, among patients with a 2-level versus single level fusion [13]. Mende et al. also found 2-level ACDF implants to subside more frequently than hybrid constructs, due primarily to reduced stress at the segments that received arthroplasty [14]. Given the positive safety and efficacy profile for mACDF procedures, the demonstrated non-inferiority for multilevel hybrid surgery in the current study is particularly meaningful and shows the value of hybrid surgery as an alternative procedure, especially given the above benefits related to range of motion and fusion-related complications [7]–[8].

We were not able to specifically assess ASD in this analysis, as we did not have information on the specific vertebrae impacted in the subsequent surgeries; however, we were able to investigate subsequent surgeries. It is worth noting that due to the limited duration of follow-up in this analysis in relation to the trajectory of revision surgeries in multilevel degenerative disc disease, this study reflects near term, post-procedural outcomes. Prior analyses have reported annual rates of ASD or subsequent surgery to address ASD of approximately 2–3% annually [3, 5]. This analysis reported rates of subsequent surgery around 2% over an average of two years; thus, our rate of subsequent surgery, which would be expected to include both ASD-related and well as non-ASD-related secondary surgeries, largely aligns with the available literature over the study trajectory.

The similar rates of 30-day hospitalizations and significantly lower rates of 90-day hospitalizations, for the hybrid surgery patients identified in this analysis also suggests that hybrid surgery patients have similar recovery post-procedure as mACDF patients. In line with these findings, multiple clinical studies have also demonstrated similar or improved clinical outcomes and recovery measures with hybrid surgery versus mACDF. [7–9] Specifically, two recent meta-analyses of hybrid surgery versus ACDF and CDA in patients with multilevel cervical degenerative disc disease found improved C2-C7 ROM and reduced adjacent segment ROM with hybrid surgery versus ACDF [15]–[16]. Like our analysis, the numbers of reported postoperative complications did not differ significantly between hybrid surgery patients vs. multilevel CDA or mACDF. Other analyses have also reported improved clinical recovery, reduced post-operative neck pain, improved C2-C7 and adjacent segment ROM, and reduced graft subsidence with hybrid surgery [17].

Our analysis also sought to provide outcomes for patients with different patterns of multi-level surgery by examining outcomes within subgroups of patients who had multilevel spinal surgery via primary or secondary procedures. Trends in patients with primary multi-level procedures largely reflected results within the full sample, consistent with their accounting for the majority of said sample. Conversely, trends for the secondary surgery sub-cohorts diverged from the other two subgroups and overall sample. The limited sample size of the secondary hybrid surgery cohort (37 patients) is likely a large contributor to these trends, which must be interpreted with caution. The duration of follow-up in this study also plays a greater role with the secondary surgery cohorts as only those patients who had maintained continuous eligibility with their insurer (average of 2-years in this analysis) between their first and second procedure would be included in our analysis. As the trajectory of secondary surgery can be notably longer than two years for many patients, it is likely that our secondary surgery patients reflect more complex cases who required multiple surgeries in quick succession.

### Limitations

The limitations of this study include those inherent in any retrospective, claims-based analysis. The MarketScan Research Databases rely on administrative claims data for clinical detail. These data are subject to data coding limitations and data entry error, and the potential for misclassification of spinal disease/procedures, covariates, or study outcomes is present. For example, codes for spinal surgery do not define the precise location of the surgery and coding for revision surgery is inconsistent, therefore it was not possible to identify the reason for, or location of, subsequent surgeries. For this reason, this study focused on surgeries that changed the underlying spinal construct to define distinct procedural episodes. Despite the limitations on clinical data, this study was able to examine near-term outcomes of hybrid surgery vs. mACDF in a more generalizable population than has previously been available to provide insight into real-world patient outcomes. It would be expected that our study sample is composed of a broader clinical population that includes patients who may not have qualified for clinical trial or observational study cohorts. Unfortunately, were not able to address other outcomes relevant to spinal surgery, such as ROM and quality of life, due to the lack of patient and clinician reported outcomes data within administrative claims. Finally, this study was limited to individuals with employer-sponsored health coverage or primary Medicare supplemental coverage, and consequently, results of this analysis may not be generalizable to patients with other insurance or without health insurance coverage.

## Conclusion

The initial goal of any new proposed treatment is to establish safety. Although several clinical studies have investigated outcomes in hybrid surgery versus mACDF, these studies have been limited both in number and sample size. This analysis utilized administrative claims data to expand assessments of the safety of hybrid surgery to a large, national population of patient receiving either hybrid surgery or mACDF as a treatment for multilevel cervical degenerative disc disease. Overall, the results of this analysis demonstrated non-inferiority of hybrid surgery compared to the current standard of care, mACDF. The established safety and efficacy record of mACDF in multilevel spinal surgery is a testament to the non-inferiority finding for hybrid surgery, as the mACDF comparator has been the standard treatment for decades. The results of this real-world analysis, in concert with previously published clinical and biomechanical studies, suggest that hybrid surgery is a safe and effective method for management of multilevel spinal disease that may help to reduce ASD by maintaining spinal mobility compared to mACDF.

## Data Availability

The data that support the findings of this study are available from Merative. Restrictions apply to the availability of these data, which were used under license for this study. The MarketScan Databases can be obtained through Merative: https://www.merative.com/contact.
